# Health-Related Internet Use and Cyberchondria in Adolescents: Population-Based Cross-Sectional Survey

**DOI:** 10.2196/65792

**Published:** 2025-11-28

**Authors:** Stefanie Maria Jungmann, Elena Dessauer

**Affiliations:** 1 Department of Clinical Psychology and Psychotherapy of Childhood and Adolescence, Johannes Gutenberg-University Mainz, Wallstraße 3, Mainz, 55122, Germany, 49 61313939201

**Keywords:** adolescents, compulsive behavior, cyberchondria, health anxiety, health-related internet search, hypochondriasis, intolerance of uncertainty, problematic internet use, teenager, young people

## Abstract

**Background:**

Health-related internet searches are widespread among the general population. Cyberchondria, that is, excessive health-related internet research that leads to emotional stress, showed significant associations with personality traits and psychological symptoms in adult samples. Although adolescents exhibit high levels of internet use, strong interest in health topics, and heightened vulnerability to anxiety symptoms and anxiety disorders, little research has examined cyberchondria in this age group.

**Objective:**

Based on findings from adult populations, this study is among the first to investigate cyberchondria in adolescents and its associations with psychological traits (intolerance of uncertainty and health anxiety) and symptoms (problematic internet use and compulsive behavior). In addition, we examined whether intolerance of uncertainty moderates the relationship between cyberchondria and health anxiety.

**Methods:**

A cross-sectional, web-based survey was conducted between April and July 2023 among adolescents (N=159; 14‐17 years, mean 15.9, SD 0.88 years; 54% male, 45% female, 1% diverse). Validated self-report questionnaires were used to assess cyberchondria, intolerance of uncertainty, health anxiety, problematic internet use, and compulsive symptoms.

**Results:**

Overall, 83% (132/159) reported searching for health-related topics on the internet. On average, they spent 8.68 minutes per day on health-related internet use (mean 8.68, SD 11.57), within a total of 238 daily minutes per day on total internet use (mean 238.3, SD 103.84). Cyberchondria showed strong positive correlations with health anxiety (*r*=0.54; *P*≤.001), problematic internet use (*r*=0.50; *P*≤.001), compulsive behavior (*r*=0.47; *P*≤.001), and intolerance of uncertainty (*r*=0.55; *P*≤.001). The moderation analysis revealed a significant overall model (*∆R^2^*=8.08%, *F*_3, 155_=38.26; *P*≤.001), but intolerance of uncertainty was not a significant moderator (*∆R^2^*<.01%, *F*_1, 155_=0.79; *P*=.38, 95% CI −0.01 to 0.03).

**Conclusions:**

The results suggest that health-related internet research and cyberchondria seem to be as relevant in adolescence as they are in adulthood, with similar associations to psychological traits and symptoms. The findings particularly support theoretical models that emphasize the role of intolerance of uncertainty and health anxiety. From a practical perspective, recognizing cyberchondria in adolescence could inform early prevention and psychoeducational strategies, especially given the high prevalence of health-related information seeking on the internet among adolescents. Further longitudinal research is needed to clarify causal pathways and to evaluate possible intervention approaches.

## Introduction

### Health-Related Internet Use and Cyberchondria: Definitions, Mechanisms, and Evidence

In the age of advancing digitalization, digital media plays a major role in everyday life. In addition to social media, internet search engines also influence the way people obtain knowledge and information. In addition to communicative and entertaining purposes, the internet is also used to obtain information on health-related topics, such as nutritional behavior, mental health, and physical fitness [1]. Health-related internet research is widespread among the general population. Up to 80% of the general adult population inform themselves about health-related content on the internet [2,3]. Health-related internet use can be beneficial—for example, by promoting health literacy—but it can also have unfavorable aspects. When people look up causes of mild, harmless, or temporary bodily symptoms on the internet (eg, headache) negative emotions (eg, health worries) may arise or be reinforced by search behavior, characteristics of the internet, the information received, and how it is processed (eg, click-driven ranking, attention-grabbing or emotional posts, and catastrophizing processing style). When health-related internet searching is repeated or excessive, accompanied by emotional distress (typically worry and anxiety) and persists despite negative consequences, this pattern is referred to as cyberchondria [4]. The term cyberchondria is made up of the words cyber for internet use and chondria for hypochondria (health anxiety). This behavior often results from the need to reduce health concerns and fears, but on the contrary, can also lead to increased anxiety and health worries [3,5]. According to McElroy and Shevlin [6], cyberchondria is multidimensional and has the following 5-dimensional structure: excessiveness (repeated and obsessive search), negative affect (subjective anxiety or burden caused by searching for health-related issues), interruption of everyday activities (impact on everyday activities caused by health-related searches), reassurance behavior (desire to consult a doctor about the information researched), and mistrust of medical professionals (the tendency to trust health-related internet research more than medical staff).

A model for the development and maintenance of cyberchondriac behavior [3] assumes that vulnerability factors (eg, increased trait health anxiety), prior learning experiences, metacognitions, and situational triggers contribute to health-related internet search, the further extent and course of which is determined by moderating factors (eg, characteristics of the search process) and subsequent conditions (eg, reduction or reinforcement of anxiety and repetition of the search). Furthermore, some studies postulate that intolerance of uncertainty could be a risk factor for cyberchondria [7,8]. Some studies in adulthood showed medium positive correlations between intolerance of uncertainty and cyberchondria (*r*=0.20-0.52) [7,9-11], and there is also evidence of a moderating effect of intolerance of uncertainty on the relationship between health-related internet research and health anxiety [9,12]. According to Fergus [9], the correlation between the frequency of health-related internet searches and health anxiety became stronger with increasing intolerance of uncertainty. These results also suggest that avoiding uncertainty is strongly associated with negative affect due to health-related internet searches [7]. According to the Intolerance-of-Uncertainty Model [13], individuals high in intolerance of uncertainty experience ambiguous or uncertain information as particularly distressing, leading to maladaptive coping behaviors. In the context of health anxiety, uncertainty about bodily symptoms or internet-sourced information may trigger excessive reassurance seeking via repeated internet searches and related channels (eg, forums and social media). However, the significance of intolerance of uncertainty on the relationship of cyberchondria to health anxiety has not yet been finally clarified [14].

Previous research has shown that cyberchondria in adulthood is significantly positively correlated with obsessive-compulsive symptoms, health anxiety, and problematic internet use in particular, whereby the highest correlation was mostly found with health anxiety [2,8,10,15-24]. Two meta-analyses found a correlation between cyberchondria and health anxiety of *r*=0.62 and *r*=0.63 [3,23].

In addition, an experimental and a longitudinal study also showed that internet searches on health-related topics and symptoms can trigger or intensify negative affect and health worries [25,26]. In the current research literature, cyberchondria is viewed transdiagnostically [1], that is, it is not specifically closely related to just one diagnosis, but probably to several psychopathological domains (such as anxiety and compulsive behavior). With regard to compulsive behaviors, it should be noted that these are explicitly considered an aspect of the cyberchondria construct and are represented as a subscale in the Cyberchondria Severity Scale (CSS) [6].

### Relevance and Previous Findings in Adolescents

Young people in particular show a high, if not the highest, level of internet use. Worldwide, around 70% of youth use the internet compared to 48% of the total population [27]. In addition to the high prevalence, around 50% of 13‐ to 17-year-olds also use the internet almost constantly [28]. In addition to benefits such as information gathering and social exchange, the internet can also bring dangers (such as overuse, anxiety, sleep problems, and negative body image) [29]. It is assumed that media and internet use can have relevant effects on the adolescent brain, neurophysiological functions, and mental health [29-31]. Overall, appropriate use of the internet is therefore also seen as an essential developmental task in adolescence [32].

Although young people spend a lot of time on the internet and health-related topics play an important role in adolescence, health-related internet searches and cyberchondria have hardly been studied in adolescence. Compared to the findings in adulthood, findings in childhood and adolescence are still largely unclear and represent an open field of research [1]. In a representative North American study, 84% of 1156 adolescents between the ages of 13 and 18 years searched for health-related information on the internet [33]. At the same time, health-related internet searches are particularly prevalent from the age of 15 years, and prevalence rates appear to increase with age [34,35]. Only recently, a few studies showed a positive correlation between cyberchondria and health worries in adolescents [36,37]. Findings from Demir et al [36] indicate that in adolescents, health anxiety can be predicted by sex, the intensity of internet searching, and the severity of cyberchondria symptoms. Köse and Murat [38] found that higher smartphone addiction among adolescents was associated with greater cyberchondria, whereas total time spent on the internet was not. When feelings of uncertainty are taken into account, one study with an adolescent sample [39] found that psychological uncertainty (social insecurity and uncertainty) mediated the relationship between cyberchondria and depressive symptoms.

Given the central role of the internet in adolescence, the early onset of anxiety symptoms in this developmental period, the research gaps on cyberchondria in this age group [36,39], this study is the first to systematically investigate health-related internet research and cyberchondria in adolescents, with a particular emphasis on the associations between cyberchondria and psychopathology. The study examines the frequency, extent, and type of health-related internet research among adolescents, as well as relationships between cyberchondria, intolerance of uncertainty, health anxiety, and psychological symptoms (obsessive-compulsive symptoms and problematic internet use). Specifically, based on previous findings (primarily from adult samples), the following hypotheses will be investigated (Textbox 1).

In order to test these hypotheses and gain deeper insight into the relationships between cyberchondria, health anxiety, intolerance of uncertainty, problematic internet use, and compulsive behavior, the following section provides a detailed description of the methodology, including the data collection process, instruments used, and statistical analysis.

Textbox 1. **Study hypotheses.**

- In adolescents, there is a significant, at least medium-strong positive correlation between cyberchondria and intolerance of uncertainty.

-  This correlation also remains when the duration of health-related internet use (in minutes) is controlled for.

- In adolescents, there is a significant, at least medium-strong positive correlation between cyberchondria and

-  health anxiety
-  problematic internet use
-  compulsive behavior
-  the correlations in health anxiety, problematic internet use, and compulsive behavior also remain when the duration of health-related internet use (in minutes) is controlled for.

- The relationship between cyberchondria and health anxiety is significantly moderated by intolerance of uncertainty.

## Methods

### Design and Recruitment

The research design is a correlational cross-sectional design using a web-based survey via the SoSci Survey platform [40]. Data collection began in mid-April 2023 and ended in mid-July 2023. The participants of the study “Health in the digital age – What are you googling? Health-related Internet research and cyberchondria among adolescents” were recruited in Germany via social media, during substitute teaching hours in schools, and via the open day of the Institute of Psychology. A flyer containing a QR code was distributed, allowing participants to access the survey directly by scanning the code with a mobile device. Participants were eligible if they were between 14 and 17 years of age and demonstrated sufficient German-language proficiency in both spoken and written comprehension. Exclusion criteria were age outside this range or insufficient German-language skills to understand and complete the survey. All questionnaires were completed in a standardized order, and responses were automatically stored on secure servers in anonymized form.

### Measures

The following questionnaires were used: the CSS-15 according to Barke et al [41] captures the construct of cyberchondria with the above-mentioned 5 subscales using 15 items on a 5-point Likert scale (1=never, 5=always; eg, excessiveness “I search for the same symptoms several times on the Internet.”). The CSS-15 showed good reliability (α=0.82) [41]. In this study, reliability was determined using Cronbach α and can be interpreted as good according to Cohen (α=0.80) [42].

The short form of the Intolerance of Uncertainty Scale (UIS) [43] was used to assess intolerance of uncertainty. This contains 18 items with 3 subscales, of which 6 items each cover 1 factor and mean values were used for calculation (response options: 0=does not suit me at all; 1=partly suits me; 2=suits me a lot; eg, “I cannot relax if I do not know what will happen tomorrow.”). In the original study by Gerlach et al [43], the UIS has an excellent internal consistency of α=0.90. In this study, we found an excellent reliability as well (α=0.93).

The Childhood Illness Attitude Scales (CIAS) [44] comprise 35 items on worries and attitudes related to health worries and behavior (1=never, 2=sometimes, 3=most of the time; eg, “Are you worried about your health?”). The German adaptation of the CIAS was validated by Woiwod [45]. This study demonstrates good reliability of the CIAS (α=0.87).

Compulsive symptoms were assessed using the Leyton questionnaire in the children’s version, which comprises 11 items, according to Bamber et al [46], based on Berg et al [47] (eg, “I moved or spoke in a particular way to avoid misfortune.”). The scale can be answered on a 4-point response scale (0=never, 1=sometimes, 2=most of the time, 3=always). The reliability can be rated as good (α=0.88).

Problematic internet use was assessed using the 5 items of the Short Compulsive Internet Use Scale (CIUS) according to Bischof et al [48] based on Meerkerk et al [49] (0=never, 4=very often; eg, “How often do you find it difficult to stop using the Internet?”). The reliability in the original study using a sample aged between 18 and 54 can be rated as good (α=0.89) [42,50]. We also found a good internal consistency (α=0.86).

### Health-Related Internet Research

The following questions were asked about health-related internet research: frequency, device use, internet services, emotional change and perceived feelings after research, physical symptoms, use for self-diagnosis, and rating of information quality (eg, “How many hours per day do you spend on the internet searching for health-related information [eg, physical symptoms, illnesses]? If you are unsure, try to estimate as best you can (15 minutes=0.25, 30 minutes=0.5, etc).” This study focuses on frequency and topics of health-related internet searches, emotional change and perceived feelings after health-related internet searches, the possibility of self-diagnosis through health-related internet searches, and difficulties in distinguishing between high-quality versus low-quality information.

### Statistical Analysis

The 3 hypotheses were tested using IBM SPSS Statistics for Windows (version 26) [51]. To ensure data quality, we applied several control procedures. First, we examined completion times using the Relative Speed Index (threshold >2; [40]) to identify implausibly fast responses. Second, we inspected the plausibility of responses and answer patterns. Participants were excluded if their data indicated careless responding, such as providing identical ratings across all items. Prior to hypothesis testing, data were screened for outliers and linearity using graphical inspection. Assumptions of normality, linearity, and homoscedasticity were examined through residual plots and Locally Estimated Scatterplot Smoothing (LOESS) smoothing and were deemed sufficiently met; due to the central limit theorem, a bivariate normal distribution was assumed [52]. For the correlational hypotheses (H1 and H2), bivariate correlations were calculated between cyberchondria (CSS-15), UIS, health anxiety (CIAS), problematic internet use, and compulsive symptoms. Overall, no Bonferroni correction was applied, as only a few correlations were directly hypothesis-driven, and we aimed to present a consistent overview of interrelations in the tables. To further examine potential sample effects, such as gender differences, a 1-way multivariate ANOVA was conducted, followed by post hoc univariate ANOVAs. For the moderation hypothesis (H3), a regression-based moderation analysis was performed to test whether intolerance of uncertainty moderated the association between cyberchondria and health anxiety. Visual inspection of scatterplots supported the assumption of linearity in these models. Across all analyses, the α level was .05. Given the directed nature of the hypotheses, 1-sided tests were applied where appropriate.

### Power

The G*Power software [53] was used to calculate the required sample size for correlation analyses. Assuming a medium effect (effect size *f*=0.25) and a significance level of α=.05 with a test power of 1–β=.80, a sample size of at least 88 people is required [42]. An effect size of *f*=0.25 was assumed, as this represents a medium effect according to Cohen conventions [42]. Given the lack of reliable prior estimates from pilot studies or meta-analyses on adolescent samples in Germany, this midpoint was considered a reasonable assumption for the sample size calculation.

### Ethical Considerations

This study was approved by the local ethics committee of the Department of Psychology at Johannes Gutenberg University Mainz (approval number: 2023-JGU-psychEK-006) and by the Supervisory and Services Directorate of Rhineland-Palatinate for recruitment in schools (AZ 217-23). Written informed consent was obtained from all participants. After reading the study information and a detailed explanation of data use and storage, participants were required to actively indicate consent by selecting the option to agree; if they selected “not agree,” participation was automatically terminated. Data were collected in anonymized form to ensure privacy and confidentiality; no personally identifying information was stored. Participants did not receive financial or other compensation for their participation.

## Results

### Sample Characteristics

Of the 184 individuals who opened the survey, 159 completed it in full. Only complete datasets were included in the statistical analyses; as specified in the study information, earlier dropouts were treated as study withdrawals (ie, withdrawals of consent), the final sample is therefore N=159 aged 14 to 17 (mean 15.9, SD 0.88) years (54.09% male, 44.65% female, 1.26% gender-diverse). The majority of 96.86% (n=154) were born in Germany, 0.63% in Austria (n=1), and 2.52% (n=4) indicated the category “other.” In total, 62.26% of young people live in rural areas (up to 2000 inhabitants, n=99) and the remaining 37.74% in urban areas (2000 inhabitants or more, n=60). Overall, 97.48% of young people (n=155) attend a grammar school, 1.89% are in vocational training (n=3), and 0.63% attend a secondary school (n=1). Of the pupils, 0.63% (n=1) are in the 8th grade, 18.87% (n=30) are in the 9th grade, the largest proportion (44.65%) are in the 10th grade (n=71), 32.70% (n=52) are in the 11th grade, and 1.26% (n=2) are in the 12th grade.

With regard to health status, 13.8% (n=22) of the participants stated that they are currently affected by a mental disorder: attention-deficit/hyperactivity disorder (n=1), anxiety disorder (n=1), depression (n=2), eating disorder (n=2), “misophonia” (n=1), and posttraumatic stress disorder (n=3). Overall, 12.96% (n=21) of the adolescents confirmed the question about physical illnesses: allergies, asthma, thoracic spine blockage, strained thigh, torn ligaments, herniated disc, chronic pain, diabetes mellitus type 1, “knee problems,” shin pain, “nose problems,” and migraine.

### Health-Related Internet Research

Among adolescents, 83.02% (132/159) reported searching the internet for health-related topics (eg, symptoms or illnesses), and 11.3% (18/159) did so often or very often (Multimedia Appendix 1). On average, adolescents spent 238.3 (SD 103.84; range 60-480) minutes per day on the internet and 8.68 (SD 11.57; range 0-60) minutes per day on health-related internet searching (Multimedia Appendix 2). The duration of general internet use (in minutes) and the duration of health-related internet searches (in minutes) were not significantly correlated (*r*=0.06; *P*=.51).

The most frequently searched health-related topics on the internet among adolescents were pain (68.94%, 91/132, eg, headache, stomach ache, sore throat, and toothache), mental health problems or disorders (15.15%, 20/132, eg, depression, burnout, and posttraumatic stress disorder), heart disease (6.82%, 9/132), coronavirus (6.82%, 9/132), and cancer (4.55%, 6/132). Multimedia Appendix 2 illustrates the perceived change in well-being or feelings after the health-related internet research. Table 1 lists the emotions experienced after the health-related internet research (multiple answers were possible, of which approximately 47% also reported “hardly any feeling”).

**Table 1.** Frequencies and percentages of perceived feelings after health-related internet research (N=132).

Perceived emotions and experience	Participants, n (%)
Worries	69 (52.27)
Hardly a feeling	62 (46.97)
Anxiety	49 (37.12)
Relief	40 (30.30)
Hope	18 (13.64)
Relaxation	17 (12.88)
Hopelessness	17 (12.88)
Sadness	14 (10.61)
Disgust	9 (6.82)
Anger	7 (5.30)
Joy	7 (5.30)
Shame	5 (3.79)
Other (eg, confusion and insecurity)	7 (5.30)

Regarding rating difficulties to distinguish between high-quality and low-quality information, the participants stated on average that they rarely or sometimes had difficulties in distinguishing (mean 1.54, SD 1.06, range 0 [never] to 4 [very often]). When asked how often young people use the internet to find the causes of symptoms and carry out self-diagnosis (response scale: 0=never, 4=very often), young people indicated a lower average frequency (mean 1.11, SD 1.04).

### Descriptive Statistics of the Used Questionnaires

Table 2 illustrates the means and SDs of the used questionnaires and their subscales.

A 1-way multivariate ANOVA revealed a significant difference between genders for the combined dependent variables Cyberchondria and its subfacets (*F*_10, 304_=3.37; *P*<.001, partial η²=.10). Post hoc univariate ANOVAs were performed for each dependent variable. The results indicate that girls have higher scores than boys in the overall scale of cyberchondria (*F*_2, 156_=4.24; *P*=.02, partial η²=.05) and in particular the dimensions of negative affect (*F*_2, 156_=9.53; *P*<.001, partial η²=.11), and excessiveness (*F*_2, 156_=4.90; *P*=.01, partial η²=.06). At the same time, there was no significant correlation between age and cyberchondria (*r*=0.09; *P*=.12).

**Table 2.** Descriptive statistics of the measurements (N=159).

Used questionnaires and their subscales		Mean (SD)	
CSS^a^		1.83 (0.54)	
Negative affect		1.88 (0.93)	
Excessiveness		2.42 (1.00)	
Distrust of medical staff		1.73 (0.93)	
Reassurance		1.86 (0.83)	
Interruption of daily activities		1.27 (0.54)	
CIAS^b^		52.20 (8.32)	
Anxiety		19.40 (5.13)	
Symptom impact		12.00 (2.67)	
Seeking help		15.63 (3.10)	
Treatment experience		5.20 (1.27)	
UIS^c^		0.75 (0.47)	
Stress		0.76 (0.53)	
Limited ability to act		0.72 (0.58)	
Vigilance		0.77 (0.49)	
Leyton^d^		9.11 (6.48)	
Obsession and incompleteness		4.10 (2.95)	
Cleanliness		3.18 (2.11)	
Compulsions		1.87 (2.53)	
CIUS^e^		1.70 (0.97)	

a CSS: Cyberchondria Severity Scale.

b CIAS: Childhood Illness Attitude Scales.

c UIS: Intolerance of Uncertainty Scale.

d Leyton: Leyton Questionnaire.

e CIUS: Compulsive Internet Use Scale.

### Relationships Between Cyberchondria and Intolerance of Uncertainty and Psychological Symptoms

Hypothesis 1 examined the relationship between cyberchondria and intolerance of uncertainty. The correlation analysis showed a significant and strong positive correlation (*r*=0.55; *P*≤.001), indicating that higher intolerance of uncertainty is strongly associated with higher levels of cyberchondria. If the subscales are examined more closely, Table 3 shows in particular strong positive correlations between the subscale negative affect of the CSS and subscales of the UIS (*r*=0.50; *P*≤.01), suggesting that greater difficulties in dealing with uncertainty are linked to stronger negative emotional reactions when searching for health information. Table 4 shows the results after controlling for the duration of the health-related internet search (in minutes). The correlation between the total values of CSS and UIS remained moderately significant, albeit somewhat lower (*r*=0.42; *P*≤.001), indicating that the association persists regardless of search duration. The subscale correlations also remained mostly significant, albeit descriptively slightly lower. When controlling for duration of use, a change was found in particular with regard to the excessiveness subscale of the CSS (only significant correlation with the subscale of the UIS “limited ability to act,” *r*=0.24; *P*<.05, meaning that difficulties in acting under uncertainty are specifically linked to more excessive search behavior).

Table 5 shows the results regarding the relationship between cyberchondria and (1) health anxiety, (2) problematic internet use, and (3) obsessive-compulsive symptoms. We found significantly strong positive relationships between cyberchondria and (1) health anxiety (*r*=0.54; *P*≤.001), (2) problematic internet use (*r*=0.50; *P*≤.001), and (3) compulsive behavior (*r*=0.47; *P*≤.001). These correlations were also evident when controlling for the duration of health-related internet use: health anxiety (*r*=0.44; *P*≤.001), problematic internet use (*r*=0.43; *P*=.001), and compulsive behavior (*r*=0.40; *P*≤.001), indicating that higher levels of these traits remain reliably associated with greater cyberchondria even after accounting for search duration.

**Table 3.** Correlations of Cyberchondria Severity Scale (CSS) and Intolerance of Uncertainty Scale (UIS) including subscales (N=159).

		1	2	3	4	5	6	7	8	9	10
1. CSS^a^ (full scale), *r*		1	0.79^b^	0.77^b^	0.36^b^	0.59^b^	0.64	0.55	0.52	0.48	0.46
2. CSS subscale: Negative affect, *r*		0.79^b^	1	0.55	0.05	0.41	0.48	0.52	0.5	0.45	0.43
3. CSS subscale: Excessiveness, *r*		0.77^b^	0.55^b^	1	0.01	0.4	0.36	0.37	0.36^b^	0.37	0.26
4. CSS subscale: Mistrust, *r*		0.36^b^	0.05	0.01	1	–0.17^c^	0.21^b^	0.3	0.3	0.23	0.28
5. CSS subscale: Reassurance, *r*		0.59^b^	0.41^b^	0.4^b^	–0.17^c^	1	0.21	0.14	0.11	0.13	0.14
6. CSS subscale: Interruption, *r*		0.64^b^	0.48^b^	0.36^b^	0.21^b^	0.21^b^	1	0.38	0.36	0.31	0.35
7. UIS^d^ (full scale), *r*		0.55^b^	0.52^b^	0.37^b^	0.3^b^	0.14^c^	0.38^b^	1	0.9	0.91	0.84
8. UIS subscale: Stress, *r*		0.52^b^	0.5^b^	0.36^b^	0.3^b^	0.11	0.36^b^	0.9^b^	1	0.75	0.64
9. UIS subscale: Limited ability to act, *r*		0.48^b^	0.45^b^	0.37^b^	0.23^b^	0.13^c^	0.31^b^	0.91^b^	0.75^b^	1	0.62
10. UIS subscale: Vigilance, *r*		0.46^b^	0.43^b^	0.26^b^	0.28^b^	0.14^c^	0.35^b^	0.84^b^	0.64^b^	0.62^b^	1

a CSS: Cyberchondria Severity Scale.

b This correlation is significant at the .01 level (1-sided).

c This correlation is significant at the .05 level (1-sided).

d UIS: Intolerance of Uncertainty Scale.

**Table 4.** Correlations of Cyberchondria Severity Scale (CSS) and Intolerance of Uncertainty Scale (UIS) including subscales after controlling for the duration of health-related internet use (in minutes).

	1	2	3	4	5	6	7	8	9	10
1. CSS^a^ (full scale), *r*	1	0.75^b^	0.69^b^	0.36	0.53	0.61	0.42	0.38	0.36	0.38
2. CSS subscale: Negative affect, *r*	0.75^b^	1	0.41^b^	0.03	0.3	0.41	0.44	0.42	0.38	0.38
3. CSS subscale: Excessiveness, *r*	0.69^b^	0.41^b^	1	0.02	0.27	0.25	0.22	0.19	0.24	0.14
4. CSS subscale: Mistrust, *r*	0.36^b^	0.03	0.02	1	–0.18	0.21	0.29	0.28	0.18	0.31
5.CSS subscale: Reassurance, *r*	0.53^b^	0.3^b^	0.27^b^	–0.18	1	0.14	0.02	–0.02	0.03	0.05
6.CSS subscale: Interruption, *r*	0.61^b^	0.41^b^	0.25^c^	0.21	0.14	1	0.28	0.26	0.23	0.27
7. UIS^d^ (full scale), *r*	0.42^b^	0.44^b^	0.22	0.29^b^	0.02	0.28^b^	1	0.89	0.91	0.85
8.UIS subscale: Stress, *r*	0.38^b^	0.42^b^	0.19	0.28^b^	–0.02	0.26^b^	0.89^b^	1	0.72	0.63
9. UIS subscale: Limited ability to act, *r*	0.36^b^	0.38^b^	0.24^c^	0.18	0.03	0.23^c^	0.91^b^	0.72^b^	1	0.65
10. UIS subscale: Vigilance, *r*	0.38^b^	0.38^b^	0.14	0.31	0.05	0.27	0.85^b^	0.63^b^	0.65^b^	1

a CSS: Cyberchondria Severity Scale.

b This correlation is significant at the .01 level (1-sided).

c This correlation is significant at the .05 level (1-sided).

d UIS: Intolerance of Uncertainty Scale.

**Table 5.** Correlations of the measurement instruments including subscales (N=159).

	1	2	3	4	5	6	7	8	9	10	11	12	13	14	15	16
1. CSS^a^ (full scale), *r*	1	0.79^b^	0.77^b^	0.36^b^	0.59^b^	0.64	0.54	0.56	0.56	0	0.1	0.47	0.45	0.31	0.42	0.5
2. CSS subscale: Negative affect, *r*	0.79^b^	1	0.55^b^	0.05	0.41^b^	0.48	0.57	0.62	0.56	0.01	0.02	0.48	0.49	0.3	0.41	0.44
3. CSS subscale: Excessiveness, *r*	0.77^b^	0.55^b^	1	0.01	0.4^b^	0.36^b^	0.38	0.36	0.43	0.05	0.06	0.39	0.39	0.3	0.3	0.46
4. CSS subscale: Mistrust, *r*	0.36^b^	0.05	0.01	1	–.17^c^	0.21^b^	0.08	0.19	0.1	–.20	0.03	0.15	0.13	0.07	0.18	0.18
5. CSS subscale: Reassurance, *r*	0.59^b^	0.41^b^	0.4^b^	–.17^c^	1	0.21^b^	0.32	0.19	0.32	0.23	0.1	0.11	0.07	0.08	0.14	0.17
6. CSS subscale: Interruption, *r*	0.64^b^	0.48^b^	0.36^b^	0.21^b^	0.21^b^	1	0.36^b^	0.42	0.39	–0.13	0.14	0.34	0.32	0.22	0.32	0.29
7. CIAS^d^ (full scale), *r*	0.54^b^	0.57^b^	0.38^b^	0.08	0.32^b^	0.36^b^	1	0.85	0.75	0.48	0.39	0.47	0.44	0.35	0.41	0.42
8. CIAS subscale: Anxiety, *r*	0.56^b^	0.62^b^	0.36^b^	0.19^c^	0.19^b^	0.42^b^	0.85^b^	1	0.56	0.07	0.17	0.53	0.47	0.37	0.49	0.41
9. CIAS subscale: Symptom impact, *r*	0.56^b^	0.56^b^	0.43^b^	0.1	0.32^b^	0.39^b^	0.75^b^	0.56^b^	1	0.13	0.22	0.52	0.54	0.35	0.41	0.48
10. CIAS subscale: Seeking Help, *r*	0	0.01	0.05	–.20^b^	0.23^b^	–0.13	0.48^b^	0.07	0.13^c^	1	0.15	–0.09	–0.12	0.01	–0.11	–0.04
11. CIAS subscale: Treatment experience, *r*	0.1	0.02	0.06	0.03	0.1	0.14^c^	0.39^b^	0.17^c^	0.22^b^	0.15^c^	1	0.1	0.1	0.04	0.11	0.17
12. Leyton^e^ (full scale), *r*	0.47^b^	0.48^b^	0.39^b^	0.15^c^	0.11	0.34^b^	0.47^b^	0.53^b^	0.52^b^	–0.09	0.1	1	0.88	0.79	0.88	0.43
13. Leyton subscale: Obsession, *r*	0.45^b^	0.49^b^	0.39^b^	0.13	0.07	0.32^b^	0.44^b^	0.47^b^	0.54^b^	–0.12	0.1	0.88^b^	1	0.53^b^	0.65	0.47
14. Leyton subscale: Cleanliness, *r*	0.31^b^	0.3^b^	0.3^b^	0.07	0.08	0.22^b^	0.35^b^	0.37^b^	0.35^b^	0.01	0.04	0.79^b^	0.53^b^	1	0.58^b^	0.28
15. Leyton subscale: Compulsions, *r*	0.42^b^	0.41^b^	0.3^b^	0.18^c^	0.14^c^	0.32^b^	0.41^b^	0.49^b^	0.41^b^	–0.11	0.11	0.88^b^	0.65^b^	0.58^b^	1	0.31^b^
16. CIUS^f^ (full scale), *r*	0.5^b^	0.44^b^	0.46^b^	0.18^c^	0.17^c^	0.29^b^	0.42^b^	0.41^b^	0.48^b^	–0.04	0.17^c^	0.43^b^	0.47^b^	0.28^b^	0.31^b^	1

a CSS: Cyberchondria Severity Scale.

b This correlation is significant at the 0.01 level (1-sided).

c This correlation is significant at the 0.05 level (1-sided).

d CIAS: Childhood Illness Attitude Scale.

e Leyton: Leyton Questionnaire.

f CIUS: Compulsive Internet Use Scale.

### Intolerance of Uncertainty as Moderator

When considering hypothesis 3, which assumes a moderating influence of intolerance of uncertainty on the relationship between cyberchondria and health anxiety, a moderation analysis with PROCESS macro (model 1) according to Hayes [54] showed a significant overall model (*F*_3, 155_=38.26; *P*≤.001) with a variance explanation of approximately 38%. This indicates that the predictors together accounted for a substantial proportion of the variance in cyberchondria. However, there was no significant moderation effect of intolerance of uncertainty on the relationship between cyberchondria and health anxiety (∆*R*^2^<.01 %, *F*_1, 155_=0.79; *P*=.38, 95% CI −0.01 to 0.03), meaning that intolerance of uncertainty did not significantly alter the strength of the association between the 2 variables. Based on the recommendations of Hayes [54], the interaction term was removed, resulting in a new model with main effects. On the one hand, there was a significant main effect of intolerance of uncertainty on cyberchondria (*B*=.40, *β*=.36; *P*≤.001), indicating that higher intolerance of uncertainty is strongly associated with higher levels of cyberchondria. On the other hand, there was a significant main effect of health anxiety on cyberchondria (*B*=.02, *β*=.34, *P*≤.001), suggesting that greater health anxiety is reliably linked to stronger cyberchondria symptoms.

## Discussion

### Overview

The aim of this study was to investigate health-related internet research and cyberchondria among adolescents. We investigated the occurrence and characteristics of health-related internet searches (self-reported emotional changes, type of emotion, differentiation of quality, and use for self-diagnosis). In addition, it was hypothesized that cyberchondria is significantly positively related to intolerance of uncertainty, health anxiety, problematic internet use, and compulsive behavior, with intolerance of uncertainty moderating the relationship between cyberchondria and health anxiety.

### Principal Results

While studies show that around 80% of adults conduct health-related internet searches [2,3], a similar proportion of young people (approximately 80%) report doing so, with approximately 11% stating often or very often. Regarding emotional changes and the perceived feelings after health-related internet research, most participants did not perceive any change in their feelings after health-related internet research (58%), and if there is a perceived change in well-being, young people are most likely to feel slightly worse (22%; 6% significantly worse). The latter is consistent with the finding that the most frequently reported emotion after internet research is worry (52%) and anxiety (37%). In addition, participants reported relief (30%), hope (14%), or relaxation (13%). These findings are consistent with explanatory models of cyberchondria that internet research can be regarded as a reassuring behavior, whereby negative affect or uncertainty can be partly reduced and partly triggered or intensified (depending on various factors such as the search results). Because intermittent negative reinforcement promotes resistance to extinction, the behavior may be maintained over time [3]. In light of the relatively low average search times (mean 9, SD 12 minutes), the results appear consistent with patterns typically observed in the general population. Within a dimensional concept of cyberchondria, this suggests that our study captures risk tendencies rather than excessive behavior, acknowledging that excessiveness represents only one of several subfacets of the construct.

According to the self-reports, young people stated that they have rarely or sometimes difficulty in distinguishing between low and high quality of information. On the one hand, this could indicate good digital skills or digital health literacy. On the other hand, self-reported confidence was asked, whereby in principle, a distinction can be made between knowledge and ability or application of knowledge under different (eg, ideal) conditions [55,56]. The application of knowledge can differ from self-report, and, additionally, the application of knowledge can be altered or limited in anxiety-ridden situations (eg, if there are health concerns during health-related internet searches). Therefore, this result should be interpreted with caution and could represent an interesting field of investigation for future studies.

In addition, adolescents tended to use the internet less for self-diagnosis. Although it is described as an increasing phenomenon for young people to self-diagnose (especially via social media), we found this to be less common, in contrast to previous studies reporting higher prevalence rates [57]. Possibly due to the fact that our study primarily focused on physical symptoms and illnesses, the increased tendency in this regard seems to relate more to mental health. In addition, “self-diagnosed” can possibly be perceived as very strong or fixed versus self-identifying or seeing oneself with symptoms of others.

With regard to age and gender effects, a heterogeneous picture emerges after considering the findings from this study and other research projects. Some studies indicate that young people older than 15 years of age show a higher level of health-related internet searches than young people younger than 15 years of age [58]. In addition, older adolescents (16‐17 years) stated that the internet is the main source for searching for health information. However, in contrast to these previous findings, the results presented here do not show a significant correlation between cyberchondria and age, which could possibly be due to the homogeneous sample (lower variance with regard to age). As only the age range between 14 and 17 years was included, the results could indicate that the differences in age effects are more likely to be found between childhood and adolescence. Considering gender, a previous study found that girls seem to tend to inform themselves more about pain and how to deal with it [58]. In our German sample, there was a significant difference between genders on the dependent variable of cyberchondria and its subfacets, particularly on the dimensions of negative affect and excessiveness. Girls tended to report higher levels of cyberchondria than boys. As this effect is rather weak [42], this should be taken into account in the interpretation. In addition, girls also showed higher levels of health anxiety, obsessive-compulsive symptoms, and intolerance of uncertainty.

Regarding the main analyses, as expected (hypothesis 1), we found a strong positive association between cyberchondria and intolerance of uncertainty. The strength of the association (*r*=0.55; *P*≤.001) is comparable or descriptively slightly higher than in adulthood [*r*=|0.20|-|0.52|; 7, 9, 10]. It is possible that a tendency toward intolerance of uncertainty can be seen as a kind of risk factor for cyberchondria and, as described above in the explanatory model, reassurance is sought through internet research. It is also possible that the large amount of information, some of which is ambiguous or frightening, triggers a stronger intolerance of uncertainty or that the 2 processes build each other up. It could be that adolescents are particularly susceptible to sensitive information, especially from the internet. Consequently, inaccurate and unreliable information and its sources on the internet can increase feelings of insecurity and anxiety [38]. The fact that this relationship persists while controlling for the duration of health-related internet research highlights the relevance of intolerance of uncertainty in relation to cyberchondria. This effect also supports previous findings from adulthood [7,9,10] and underlines the need for preventive interventions aimed at reducing intolerance of uncertainty.

In addition, there were also significant positive correlations between cyberchondria and health anxiety, obsessive-compulsive symptoms, and problematic internet use, with the highest association with health anxiety, consistent with studies in adults [3,23]. There is probably a vicious circle of health anxiety and cyberchondriac behavior [3,59]. A recent study on the relationship between cyberchondria and pathological health anxiety (hypochondriasis) showed that a multidimensional view of cyberchondria seems useful, as some subfacets of cyberchondria are probably more closely associated with hypochondriasis (eg, negative affect after searches) while other subfacets play a more transdiagnostic role (eg, excessiveness and characteristics of the internet) [60].

As in studies with adults, positive moderate and strong correlations were found between cyberchondria and obsessive-compulsive symptoms [10,15,61]. In contrast to adulthood, we found the strongest correlation between the CSS subscale negative affect and obsessions and incompleteness of the Layton Scale at the subscale level. The more obsessions (eg, feeling guilty) adolescents reported, the stronger the perceived negative affect after internet searches. It is possible that this close link could be explained by dysfunctional metacognitions, which play a relevant role in both obsessive-compulsive disorder symptoms and cyberchondria [10,59,62]. Overall, as already noted in the introduction, compulsiveness is also an aspect of the conceptualization of cyberchondria and is represented in a subfacet of the CSS, reflecting this overlap [6,41].

As expected, we found medium and strong relationships between problematic internet use (in terms of controllability, negative effects on sleep, affect, and social relationships) and cyberchondria, with the highest correlation with the CSS subscales negative affect and excessiveness. Also in this domain, controlling for the time spent on the internet, the associations remain relatively stable. This is consistent with Köse and Murat [34], who found that greater smartphone addiction during adolescence was associated with higher cyberchondria, whereas total time spent on the internet was not.

In addition, the Mistrust of Medical Professionals subscale merits brief consideration. Previous studies have questioned its validity, and in some cases recommended excluding it [6,16,41], and our findings in adolescents point in the same direction. The subscale showed the weakest and partly negative correlations with other subscales and no significant association with health anxiety, which may reflect methodological issues (inverse wording) or content-related difficulties, particularly among adolescents. These results suggest that the recommendation to exclude this subscale also applies to adolescent samples.

Although the moderation analysis of hypothesis 3 showed no significant influence of intolerance of uncertainty on the relationship between health anxiety and cyberchondria, the significant overall model with a high variance explanation indicates the relevance and possibly the complexity of the interaction between the constructs. It is possible that other factors, such as metacognition, play a significant role. For example, the study by Nadeem et al [63] suggests a moderating influence of metacognitions on the relationship between cyberchondria and health anxiety in adults. According to the findings of the study by Nadeem et al [63], metacognitions and health anxiety can also be regarded as significant predictors of cyberchondria. Furthermore, compulsiveness is part of the definition of cyberchondria and is also taken into account in the operationalization of cyberchondria [41]. The heterogeneity in the findings is also consistent with the current research literature [7,8]. The fact that intolerance of uncertainty is a moderator in adulthood [9] but not in adolescence may indicate that other moderators may play a more relevant role in adolescence.

### Limitations

First of all, the present sample is only partially representative, as most of the participants attended grammar schools (ie, high school education). The attempt to recruit participants from other secondary schools, vocational schools, and youth centers proved to be very challenging (requests were rejected due to structural conditions, for example). The level of education could be important when investigating cyberchondria in young people, as people with a higher level of education, for example, tend to have better access to health information. Therefore, information could possibly be better understood and interpreted, so that the credibility of internet sources is also questioned (see also findings on the distinction between low- and high-quality information). In addition, the consideration of cultural backgrounds is also a relevant aspect. The approach to health and illness, stigmatization due to illness and access to health information and services could vary depending on the culture (or, more generally, environments and learning experiences). Consequently, it seems reasonable to consider a variety of cultural backgrounds and educational levels in future research projects. A number of aspects can also be highlighted with regard to the various measurement instruments. Questionnaires and the possibility of retrospectively recording cyberchondria are particularly relevant in this context. The retrospective survey, which records past experiences in dealing with cyberchondria, offers researchers the opportunity to identify and investigate long-term patterns in dealing with cyberchondria. Ecological momentary assessment studies are particularly important for minimizing memory bias [64]. These regularly record specific behaviors and experiences in the participants’ daily lives [64]. Due to classroom testing, social desirability biases cannot be excluded. Although largely validated and standardized questionnaires were used, the CSS [41] and the UIS [43], for example, have not yet been validated on a sample of adolescents. Based on the results presented here, these measurement instruments could be validated and standardized on a larger sample of adolescents. Overall, due to the correlational cross-sectional design, we cannot draw any causal conclusions and have made this clear at points in the Discussion (possibly reciprocal relationships and vicious circles).

### Practical Implications and Future Directions

The construct of cyberchondria is a relatively new field of research in adolescence, meaning that specific mechanisms and correlations are still unexplored. Based on these findings, further research should examine which specific content adolescents seek and for what purposes they search for health-related information on the internet (eg, information, communication, or social exchange). It also remains to be seen which mechanisms can explain why some young people feel burdened by health-related internet searches and others do not. Potential moderating influences represent an important research gap. If correlations with metacognitions, emotion regulation strategies, resilience, and coping factors can be identified, it would seem sensible to derive possible interventions based on this. These implicit recommendations for action from the findings to date could aim to develop interventions that focus not only on cyberchondria itself, but also on the underlying coping mechanisms in order to achieve long-term positive effects on mental health.

In this context, Newby and McElroy [65] highlight that internet-based cognitive behavioral therapy is useful in the treatment of cyberchondria in the context of hypochondriasis. To reduce cyberchondria, it may help to limit health-related internet use in terms of time, situation, or both (eg, fewer minutes per day) and to rely on reputable sources. In the case of existing mental disorders (eg, illness anxiety disorder and obsessive-compulsive disorder), it is important to integrate the reduction of cyberchondria into a comprehensive treatment plan.

Finally, researchers should continue to investigate the short- and long-term advantages and disadvantages of health-related internet research in the future. The extent to which children and adolescents represent a particularly vulnerable group should also be analyzed in the future. Preventive measures could also be taken at an early stage, for example, in the sense of mental health literacy, so that children and young people learn to recognize when worries are heightened by which behavior and from whom or which service providers they can receive support in this case.

### Conclusion

In sum, we found, comparable with studies in adults, that the more adolescents tend to be intolerant of insecurity, the more they exhibit cyberchondriac behavior. We were also able to confirm strong positive correlations between cyberchondria and health anxiety in particular, as well as symptoms of obsessive-compulsive disorder and problematic internet use. Together with the finding that internet use can lead to emotional effects that can represent both worry and relief, this study shows that the findings from adolescence are also consistent with models of the development and maintenance of cyberchondria [3,59]. However, it is important to note that the associations found in this study do not imply causation. Future studies should further investigate the mechanisms that contribute to the development and maintenance of cyberchondria among adolescents in order to derive and develop prevention and intervention strategies better tailored to this age group. In particular, a similar phenomenon has become apparent in social media in recent years, with young people increasingly seeing or identifying with psychological symptoms reported by others. The consequences of the phenomenon and overlaps with cyberchondria remain to be investigated.

## Supplementary material

10.2196/65792 Multimedia Appendix 1 Frequency of health-related internet research (N=159, "Are you looking for health-related information on the internet [eg, body symptoms and diseases]?").

10.2196/65792 Multimedia Appendix 2 Emotional change after health-related internet research (N=132, "When you search for body symptoms or illnesses on the Internet, do you feel better or worse afterwards or is there no emotional change?"; the term "significantly better" was not stated).

## Abbreviations

CIAS: Childhood Illness Attitude Scales
CIUS: Compulsive Internet Use Scale
CSS: Cyberchondria Severity Scale
LOESS: Locally Estimated Scatterplot Smoothing
UIS: Intolerance of Uncertainty Scale
