# Supplementary figures and images for: Health-Related Internet Use and Cyberchondria in Adolescents: Population-Based Cross-Sectional Survey
Source: J Med Internet Res. 2025 Nov 28;27:e65792. doi: 10.2196/65792 (PMC12669920; doi:10.2196/65792)

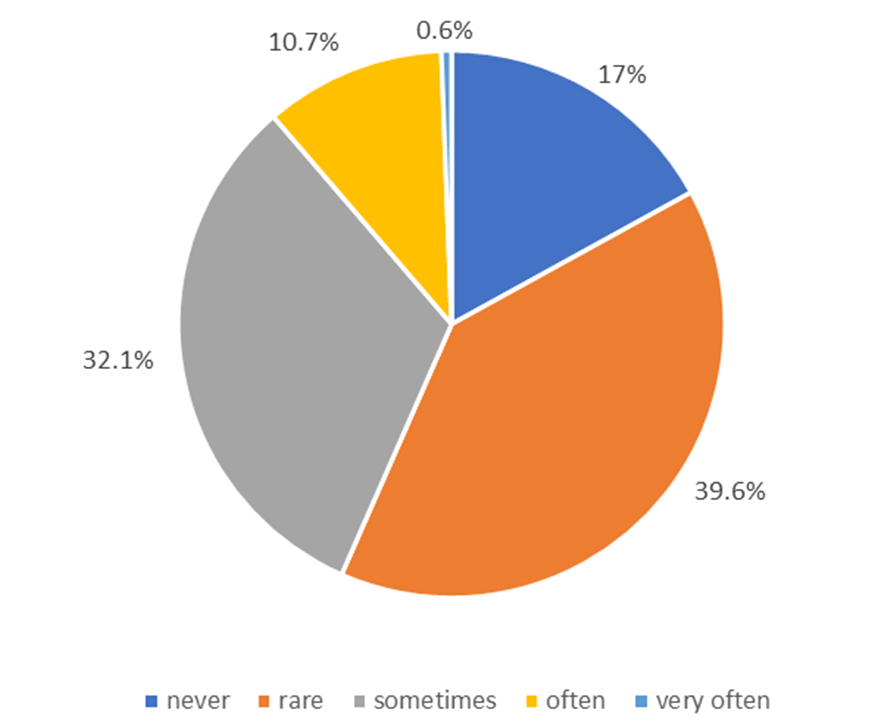

Supplement: Multimedia Appendix 1 [file jmir-v27-e65792-s001.png]

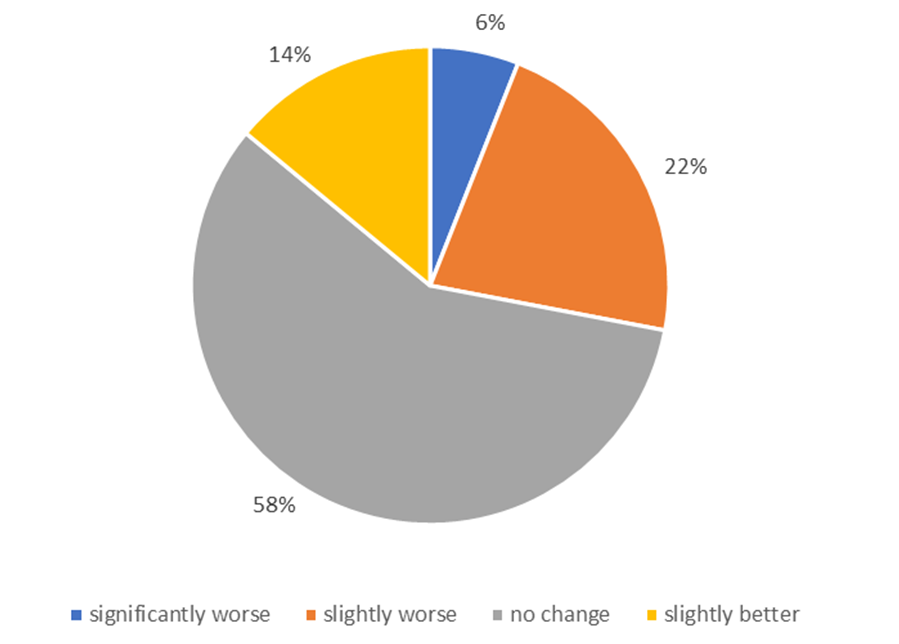

Supplement: Multimedia Appendix 2 [file jmir-v27-e65792-s002.png]
